# Image-Derived Input Functions for Quantification of A_1_ Adenosine Receptors Availability in Mice Brains Using PET and [^18^F]CPFPX

**DOI:** 10.3389/fphys.2019.01617

**Published:** 2020-01-29

**Authors:** Xuan He, Franziska Wedekind, Tina Kroll, Angela Oskamp, Simone Beer, Alexander Drzezga, Johannes Ermert, Bernd Neumaier, Andreas Bauer, David Elmenhorst

**Affiliations:** ^1^Institut für Neurowissenschaften und Medizin (INM-2), Forschungszentrum Jülich, Jülich, Germany; ^2^Department of Neurophysiology, Institute of Zoology (Bio-II), RWTH Aachen University, Aachen, Germany; ^3^Department of Nuclear Medicine, University Hospital of Cologne, Cologne, Germany; ^4^Institut für Neurowissenschaften und Medizin (INM-5), Forschungszentrum Jülich, Jülich, Germany; ^5^Neurological Department, Medical Faculty, Heinrich Heine University Düsseldorf, Düsseldorf, Germany; ^6^Division of Medical Psychology, University of Bonn, Bonn, Germany

**Keywords:** image-derived input function, positron emission tomography, A_1_ adenosine receptors, [^18^F]CPFPX, mice brains

## Abstract

**Purpose:**

*In vivo* imaging for the A_1_ adenosine receptors (A_1_ARs) with positron emission tomography (PET) using 8-cyclopentyl-3-(3-[^18^F]fluoropropyl)-1-propylxan- thine ([^18^F]CPFPX) has become an important tool for studying physiological processes quantitatively in mice. However, the measurement of arterial input functions (AIFs) on mice is a method with restricted applicability because of the small total blood volume and the related difficulties in withdrawing blood. Therefore, the aim of this study was to extract an appropriate [^18^F]CPFPX image-derived input function (IDIF) from dynamic PET images of mice.

**Procedures:**

In this study, five mice were scanned with [^18^F]CPFPX for 60 min. Arterial blood samples (*n* = 7 per animal) were collected from the femoral artery and corrected for metabolites. To generate IDIFs, three different approaches were selected: (A) volume of interest (VOI) placed over the heart (cube, 10 mm); (B) VOI set over abdominal vena cava/aorta region with a cuboid (5 × 5 × 15 mm); and (C) with 1 × 1 × 1 mm voxels on five consecutive slices. A calculated scaling factor (α) was used to correct for partial volume effect; the method of obtaining the total metabolite correction of [^18^F]CPFPX for IDIFs was developed. Three IDIFs were validated by comparison with AIF. Validation included the following: visual performance; computing area under the curve (AUC) ratios (IDIF/AIF) of whole-blood curves and parent curves; and the mean distribution volume (*V*_T_) ratios (IDIF/AIF) of A_1_ARs calculated by Logan plot and two-tissue compartment model.

**Results:**

Compared with the AIF, the IDIF with VOI over heart showed the best performance among the three IDIFs after scaling by 1.77 (α) in terms of visual analysis, AUC ratios (IDIF/AIF; whole-blood AUC ratio, 1.03 ± 0.06; parent curve AUC ratio, 1.01 ± 0.10) and *V*_T_ ratios (IDIF/AIF; Logan *V*_T_ ratio, 1.00 ± 0.17; two-tissue compartment model *V*_T_ ratio, 1.00 ± 0.13) evaluation. The A_1_ARs distribution of average parametric images was in good accordance to autoradiography of the mouse brain.

**Conclusion:**

The proposed study provides evidence that IDIF with VOI over heart can replace AIF effectively for quantification of A_1_ARs using PET and [^18^F]CPFPX in mice brains.

## Introduction

The A_1_ adenosine receptors (A_1_ARs) are involved in various neurological as well as psychiatric disorders (Paul et al., 2011), and play significant role in processes such as sleep–wake regulation (Porkka-Heiskanen and Kalinchuk, 2011) and memory consolidation (Gessi et al., 2011; Paul et al., 2011). At present, multiple antagonists, agonists, and allosteric modulators are under development to explore the therapeutic effect of adenosine receptors (Kiesman et al., 2009). Small animal imaging could simplify the evaluation process of these compounds; moreover, various mice models of adenosine-related diseases might help to identify potential applications (Elmenhorst et al., 2013). Therefore, there is a high interest in *in vivo* imaging techniques for the A_1_AR with positron emission tomography (PET) in mice to visualize molecular processes quantitatively. PET with the radiotracer 8-cyclopentyl-3-(3-[^18^F]fluoropropyl)-1-propylxanthine ([^18^F]CPFPX) can be used to quantify the *in vivo* concentration of A_1_ARs in the brain (Bauer et al., 2003). For this quantification, the concentration of parent radiotracer in plasma is necessary as the input function to the brain. The arterial input function (AIF) is still the gold standard (Meyer et al., 2006; Chen et al., 2007; Zanotti-Fregonara et al., 2009a) for quantification of target receptors via the invasive procedure of arterial cannulation. However, in mice, the total blood volume is small, and continuous blood sampling during the whole experiment is relatively difficult and technically challenging (Laforest et al., 2005; Yee et al., 2005). In addition, measuring the AIF might affect the physiological function of the small animal. Multiple blood samplings in mice have often conducted as a terminal procedure preventing longitudinal studies within individual (Kim et al., 2006; Ferl et al., 2007; Wu et al., 2007).

Several methods have therefore been developed to overcome the difficulties of arterial blood sampling including image-derived input functions (IDIFs) (Chen et al., 1998; Naganawa et al., 2005; Su et al., 2005; Fang and Muzic, 2008; Mourik et al., 2008), which became an attractive non-invasive alternative compared to AIF. The high spatial resolution of modern small-animal PET scanners (Lecomte et al., 1994; Cherry et al., 1997; Chatziioannou et al., 1999) allows to acquire IDIFs even from small anatomical regions. In brain PET studies from mice, only a few organs such as heart and abdominal vena cava/aorta are expected to be directly visible from initial time frames after injection. Accordingly, IDIFs were successfully established in “large” (∼2 mm diameter) blood vessels in mice (Lanz et al., 2014).

Most studies, however, reported the extraction of the IDIFs from rodent hearts because outlining blood vessels was challenging (Parker and Feng, 2005; Zanotti-Fregonara et al., 2009b; Croteau et al., 2010). When using heart muscle cells or liver, however, the spill-over effects have to be considered. Compared with heart, IDIFs from “large” blood vessel are less influenced by spill-in effects from surrounding tissues (Lanz et al., 2014), while partial volume effects caused by limited resolution and difficult delineation have to be considered carefully.

According to the previous results and the specific distribution pattern of A_1_AR, we extracted IDIFs over both heart as well as abdominal vena cava/aorta in mice scanned with PET and [^18^F]CPFPX. For quantification of A_1_AR availability in mice brains, three different approaches for volume of interest (VOI) definition for respective IDIFs were selected.

Since the radioligand we used in this study showed fast metabolization, namely, the parent radioligand accounted for only ∼18% of total radioactivity in whole blood 60 min after radiotracer injection, data were corrected for metabolites.

To evaluate the accuracy and precision of the different image-derived input approaches, AIFs from the same animals were used as reference standard.

Thus, the aim of this present study was to define appropriate IDIFs for dynamic [^18^F]CPFPX PET in mice.

## Materials and Methods

### Animal Preparation

All procedures were approved by German regional authorities (Landesamt für Natur, Umwelt und Verbraucherschutz) and performed on the basis of the German Animal Welfare Act. The animal experimental data reported in this study are in compliance with the Animal Research: Reporting *in vivo* Experiments guidelines. Five healthy male C57BL/6 mice (28 weeks; 37.80 ± 5.42 g, mean ± SD) with free access to standard mouse food and water were housed in a 12 h light/dark cycle at 22°C.

Anesthesia was introduced with 5% isoflurane in 2 L/min oxygen and maintained at 1.5–2% isoflurane in 1 L/min oxygen. The surgical cannulation (PE10, Becton Dickinson, Sparks, MD, United States) of the femoral artery and heparin-coated microtubes served for blood sampling; another catheter placed in the tail vein was used for a bolus injection of [^18^F]CPFPX. The dead volume of the catheter was 14.17 ± 2.09 μl.

Breathing rate (pressure pad, 41 ± 10 bpm) and body temperature (rectal probe) were used to continuously monitor all animals throughout the PET scans (BioVet System, m2m Imaging, Salisbury, QLD, Australia). A constant body temperature (37.17 ± 0.58°C) was maintained by a heating lamp.

### [^18^F]CPFPX PET Scan and Image-Derived Input Function Extraction Methods

Radiosynthesis and formulation of [^18^F]CPFPX were performed as described earlier (Holschbach et al., 2002). [^18^F]CPFPX was routinely obtained ready for injection with a radiochemical yield of 20 ± 5%, a radiochemical purity of 99.5 ± 0.3%, and a molar activity of 396 ± 114 GBq/mol.

All PET data were acquired on a Siemens Inveon Multimodality PET scanner (Siemens, Knoxville, TN, United States) with Inveon Acquisition Workplace 1.5 (Siemens). The mice were positioned supine (to allow blood sampling) with their heads fixed by the nose cone of the anesthesia system.

A dual-source ^57^Co transmission scan was processed for 10 min to correct attenuation before emission scans. Directly afterward, a bolus of [^18^F]CPFPX (0.83 ± 0.18 MBq) was injected over 1 min via a syringe pump (model 44, Harvard Apparatus, Holliston, MA, United States) at time of scan start.

List-mode PET data were acquired for 60 min after tracer application and framed into a dynamic sequence of 12 × 10, 3 × 20, 3 × 30, 3 × 60, 3 × 150, and 9 × 300 s frames. One hundred fifty-nine slices were reconstructed by filtered back projection (Ramp filter, cutoff = 0.5) after Fourier rebinning into 2D sinograms. All PET images were corrected for attenuation and scatter radiation.

**FIGURE 1:**
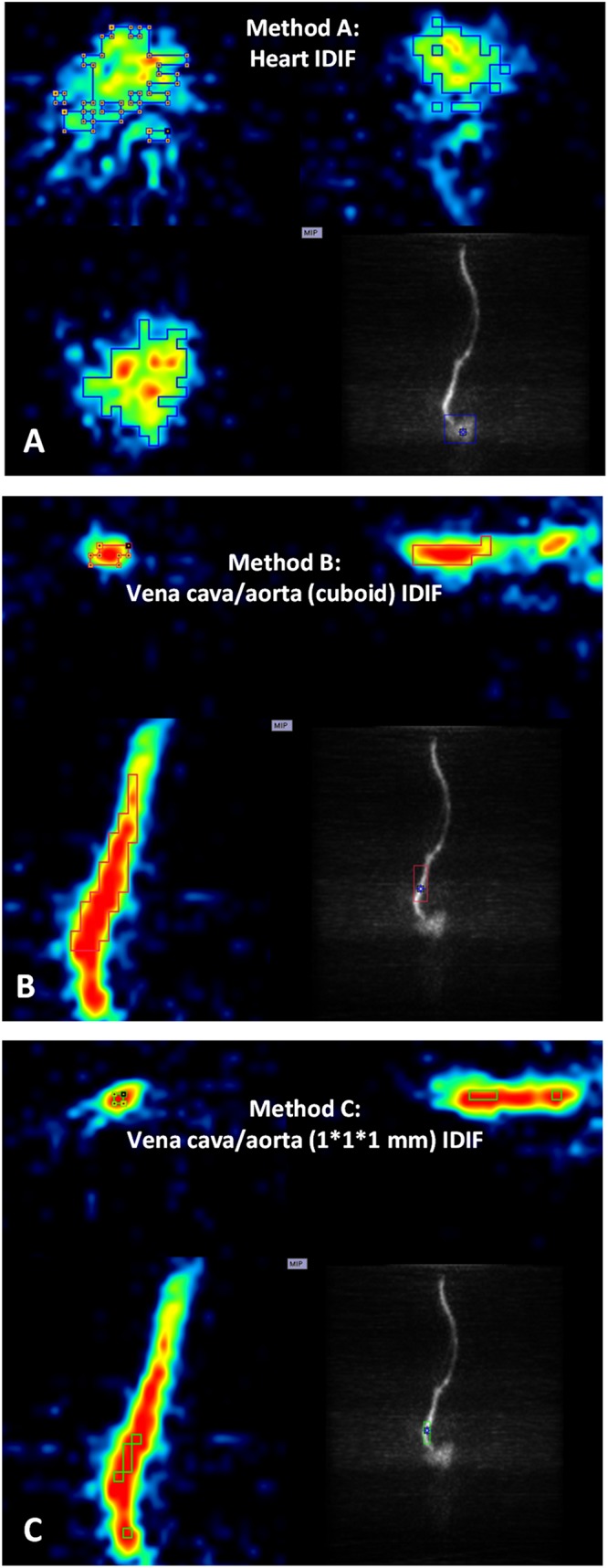
Positron emission tomography images of heart and abdominal vena cava/aorta in a representative mouse during the first 10 s after [^18^F]CPFPX bolus injection were used to define different volumes of interest ((VOIs) with image-derived input functions (IDIFs). Each image has three planes: coronal plane (upper left), sagittal plane (upper right), and horizontal plane (lower left). The lower right of each image shows a maximum-intensity-projection (MIP) map on the coronal plane. **(A)** VOI placed over the heart (cube, 10 mm). **(B)** VOI placed over abdominal vena cava/aorta region (cuboid, 5 × 5 × 15 mm). **(C)** VOI (1 × 1 × 1 mm voxels with five consecutive slices) delineated on abdominal vena cava/aorta region (centered on the highest activity spot).)

Postprocessing of PET images and activity extraction from the VOIs were performed with PMOD 3.408 software (version 3.408, PMOD Group, Zurich, Switzerland). To explore an appropriate image-derived input, three different approaches were selected (Figure 1) using the first time frames after [^18^F]CPFPX injection (image of the first 10 s) as a guide. For method A (Figure 1A), a large VOI covering the whole organ was placed over the heart (cube, 10 mm), and for method B (Figure 1B), over the abdominal vena cava/aorta region (cuboid, 5 × 5 × 15 mm). In a second step, an automatic algorithm from PMOD was utilized to identify only those voxels, within these volumes exceeding 50% of the total activity inside the VOI for subsequent read out. For method C (Figure 1C), 1 × 1 × 1 mm voxels on five consecutive slices (Lanz et al., 2014) were manually placed over the abdominal vena cava/aorta region (also centered on highest activity spot).

In this study, an optimal calculated scaling factor (α) was calculated to correct the partial volume effects of regions for IDIF effectively using the equation below:

(1) $$\text{Sum}=\sum_{i=1}^{7}{\left[C_{\text{AIF}}\,\left(t_{i}\right)-C_{\mathrm{raw}\,\_\,\mathrm{IDIF}}\,\left(t_{i}\right)\times \alpha \right]}^{2}$$

where α is the scaling factor used in this study for the three different approaches, *C*_AIF_ is the radioactivity concentration (kBq/cc) of AIF at any time point (*t*_*i*_), and *C*_raw___IDIF_ is the raw radioactivity concentration of the three different IDIFs used in this study at the same time points. The optimal scaling factor α is obtained by minimizing Sum, namely, the difference between arterial inputs and corrected image-inputs is lowest with the optimized α. More detailed information about Eq. (1) is discussed in section “Discussion.”

### Arterial Blood Sampling and Metabolite Correction

During PET acquisition, blood samples (20.51 ± 5.78 μl per time point) were taken from the femoral artery at 1, 10, 20, 30, 40, 50, and 60 min after tracer injection. After blood sampling, heparinized saline solution was used to prevent coagulation inside of the catheter by flushing. All whole-blood and plasma samples were weighed in preweighted tubes, then measured in a high-sensitivity γ-counter (ISOMED 2100; Medizintechnik Dresden, Germany) to calibrate the activity concentration with radioactive decay correction (relative to the start of the acquisition). The fraction of unchanged radioligand in total plasma activity (parent curve) was determined using a previously published approach (Meyer et al., 2004) using thin layer chromatography.

Since the radiometabolite fraction of [^18^F]CPFPX is high (Bier et al., 2006; Matusch et al., 2006), data were corrected for metabolism, and the extraction of the parent radioligand was corrected for residual activity in the precipitate after protein extraction (extraction correction). The following equation was used for data interpolation:

$$\mathrm{Total}\,\mathrm{metabolite}\,\mathrm{correction}\,\left(t\right)=\frac{1}{{\left(1+a\times {\left(t-d\right)}^{b}\right)}^{c}}$$

(2) $$\times \frac{1}{{\left(1+e\times t^{2}\right)}^{f}}$$

where *a*, *b*, *c*, and *d* are fitted to describe the metabolite fraction; *e* and *f* are fitted to describe the extraction fraction. More detailed information about Eq. (2) is discussed in section “Discussion.”

### Accuracy of Image-Derived Input Functions

The accuracy and precision of the different image-derived extraction methods of input functions were evaluated by comparison with AIF.

#### Visual Comparison

After using the scaling factor on image inputs, time–activity curves of whole-blood and parent tracer obtained with each method were compared with standard arterial whole-blood and parent time–activity curves. The comparison considered overall shape of the curves, the height of the peaks, as well as the slope of the tails.

#### Area Under Curve Ratios

In addition to the visual comparison, a quantitative analysis was performed using area under curve (AUC) ratios between the image- and arterial-derived curves (Zanotti-Fregonara et al., 2011). The image/arterial ratios of both whole-blood time–activity curves and metabolite-corrected parent time–activity curves were calculated. The image-derived parent curve of each mouse was estimated by multiplying the image-derived whole-blood time–activity curve with group average parent/whole blood ratios at the same time points.

#### Kinetic Modeling

After postprocessing with scaling factor α, PET data were analyzed with both the Logan plot model (Logan et al., 1990) and the two-tissue compartment model (2TCM) (Leenders et al., 1990; Koeppe et al., 1991). Logan analysis was implemented with *t*^∗^ = 20 min and with no weighting. 2TCM analysis used “prescribed weighting” (standard deviation of the pixel values in the VOI is used for the calculation of the weights) in all IDIF methods. For AIF, the three-term exponentials function was applied in the configuration of blood activity fitting and interpolation.

All distribution volumes (*V*_T_) with different input functions were obtained for brain regions, such as cortex, hippocampus, and thalamus for each mouse. The mean *V*_T_ ratios (image-derived input/arterial input) based on three different IDIFs were calculated and compared. The linear regression was used to show the correlation between image-derived input and arterial input, while the Bland-Altman plot was introduced to describe agreement between image-derived input and arterial input.

### Parametric Images and Autoradiography

Average parametric images (*n* = 5) of *V*_T_ in mice brains were calculated by application of Logan plot for both AIF and IDIF over heart (method A). Logan plot was set up with *t*^∗^ = 20 min, and with no masking.

The results of *in vivo* binding by different PET input functions were further verified by *in vitro* autoradiography. After decapitation, the mouse brain was removed, frozen in 2-methylbutane (−40°C) and stored at −80°C. Brain sections (20-μm thickness) were mounted on slides. Preincubation was conducted in 170 mM Tris–HCl buffer (pH 7.4) and 2 U/L adenosine deaminase for 15 min at 4°C. Later on, main incubation lasted for 2 h at room temperature with the same buffer including [^3^H]CPFPX (0.99 nM; molar activity, 2,009 GBq/mmol) (Holschbach et al., 2003), 100 μM Gpp(NH)p and 2 U/L adenosine deaminase. After washing with preincubation buffer and a rapid rinse in ice-cold water, sections were dried with a stream of air (room temperature) and exposed against phosphor-imaging plates (BAS2025; Fuji, Japan) with tritium activity standards (Amersham Biosciences, Piscataway, United States). For further processing of digital autoradiography, an image plate reader (spatial resolution of 50 μm; BAS 5000; Fuji, Japan) and image analysis software (Image Gauge 4.0; Fuji, Japan) were used. To get less background noise image in Image Gauge 4.0, 32-color type (displays in 32 pseudo-colors) was used with linear method (adjusts contrast with a linear curve).

## Results

### Blood Sample Analysis

The whole-blood activity curves reached at highest value at ∼60 s among the seven blood sampling time points, followed by a rapid drop (Supplementary Figure 1A). Plasma activity showed a stable percentage of whole-blood activity with a mean plasma/whole blood ratio of 1.67 ± 0.04. Metabolite correction of [^18^F]CPFPX was done (Eq. 2) with parameters set to *a* (0.18), *b* (1.84), *c* (0.34), *d* (0.30), *e* (0.19), and *f* (0.02). Group mean parent/whole blood ratio [(plasma/whole blood ratio) × total metabolite correction] over time was calculated (Supplementary Figure 1B) based on Eq. (2).

### Visual Analysis

**FIGURE 2:**
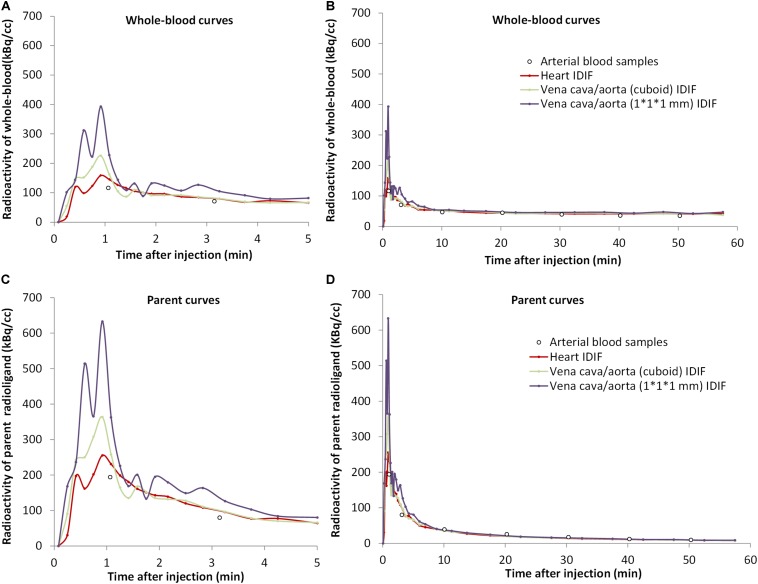
The concentrations over time of [^18^F]CPFPX in whole-blood **(A,B)** and parent radioligand **(C,D)** (with metabolite correction) curves from the arterial input function (white dots) and from the three image-derived input functions (IDIFs) with different volumes of interest (VOIs) (colored lines) in a representative animal. The left curves **(A,C)** are excerpts of the first 5 min of the total curves **(B,D)**.

After extraction of IDIFs with different methods, a calculated scaling factor (α = 1.77) was used to correct for partial volume effects in whole-blood curves (Figure 2A).

The tails of whole-blood curves obtained by three different IDIF approaches generally matched closely with the reference arterial inputs. The image-derived whole-blood curve with the VOI over the heart performed best.

Compared with the arterial parent compound curves (Figure 2B), the image-derived parent compound curves performed equally well. The shape of the image-derived input over heart (method A) was smoother than vena cava/aorta (cuboid) VOI (method B) and 1 × 1 × 1 mm VOI (method C).

### Ratios of Area Under Curves

After scaling, the mean AUC ratio between image-derived and arterial inputs of both whole-blood and corrected parent curves were estimated. As given in Table 1, the mean AUC ratio was smaller for heart (method A) and aorta (1 × 1 × 1 mm) (method C) VOIs than for aorta (cuboid) VOI (method B). Specifically, the difference in the whole-blood arterial AUC and image-derived AUC was ≤10% for both methods A and C. Moreover, method A had a whole-blood AUC ratio (1.03) that was closest to identity with a low standard deviation (SD) of 0.06.

**TABLE 1:** Mean AUC ratios (IDIF/AIF) for each VOI related IDIF in both whole-blood and parent curves.

		**Method B**	**Method C**
	**Method A**	**Vena cava/aorta**	**Vena cava/aorta**
	**Heart**	**(cuboid)**	**(1 × 1 × 1 mm)**
	**(mean ± SD)**	**(mean ± SD)**	**(mean ± SD)**
Whole-blood AUC ratio	1.03 ± 0.06	1.11 ± 0.14	1.08 ± 0.10
Parent curve AUC ratio	1.01 ± 0.10	1.04 ± 0.14	1.06 ± 0.23

**n* = 5. AUC, area under curve; VOI, volume of interest; IDIF, image-derived input function; AIF, arterial input function.*

On the other hand, the AUC ratio of parent curve in the aorta (1 × 1 × 1 mm; method C) was worse than in the heart (method A) and cuboid aorta (method B) VOIs (Table 1). The high ratio and SD in method C (1.06 ± 0.23) indicated that the results from this image-derived approach were inconstant and not fitted ideally among all tested mice, while method A performed best with an AUC ratio close to 1 and a low SD value (1.01 ± 0.10).

### Kinetic Modeling (Logan and 2TCM Analysis)

**FIGURE 3:**
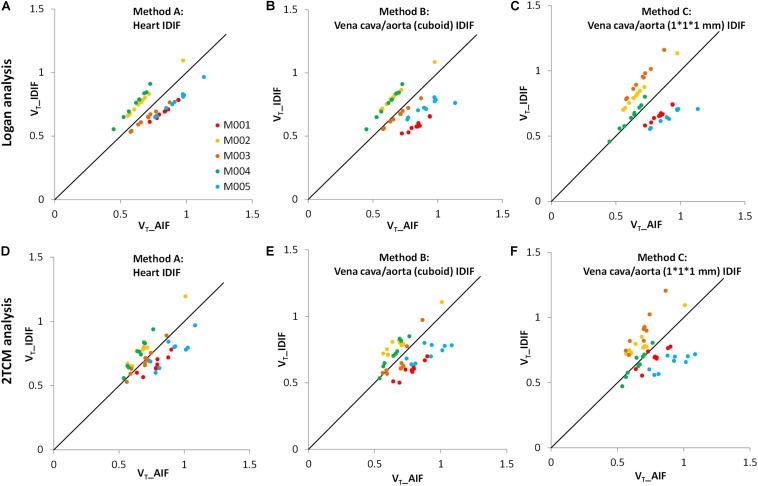
Comparison of distribution volumes (*V*_T_) values between image-derived input function (IDIF) and arterial input function (AIF). All *V*_T_ values of different input functions in various brain regions were generated by two kinetic models: the Logan graphical model (upper) and the 2TCM model (lower). **(A,D)** The IDIF with the volume of interest (VOI) placed over the heart (method A); **(B,E)** the IDIF with the VOI set over abdominal vena cava/aorta region with a cuboid (method B); **(C,F)** the IDIF with the VOI delineated with 1 × 1 × 1 mm voxels on five consecutive slices over the abdominal vena cava/aorta region (method C). Different dots represent different brain regions (*n* = 9). Different colors represent different mice (*n* = 5). The solid line is the line of identity.

In Figure 3, the *V*_T_ values of image-derived and arterial inputs generated by both Logan graphical and 2TCM analysis are presented for the following brain regions: striatum (STR), cortex (CTX), hippocampus (HIP), thalamus (THA), cerebellum (CB), hypothalamus (HYP), amygdala (AMY), olfactory bulb (OLF), and midbrain (MID).

The *V*_T_ values of each mouse obtained by the Logan graphical analysis (Figures 3A–C) show better correlations between arterial and image-derived inputs *V*_T_ in all three IDIF extraction methods, while the *V*_T_ values with the 2TCM model were more dispersed around the identity line (Figures 3D–F).

**FIGURE 4:**
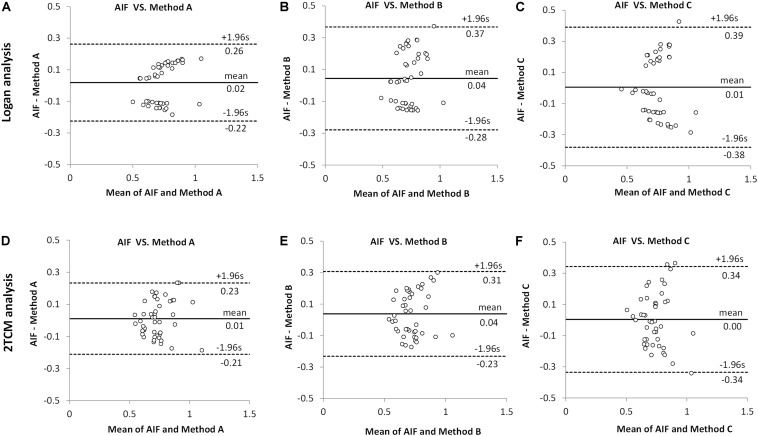
Comparison between arterial input function (AIF) and image-derived input function (IDIF) of distribution volumes (*V*_T_) by Bland–Altman analysis. All *V*_T_ values obtained by Logan and 2TCM kinetic modeling were the same as the data in Figure 3. **(A,D)** The IDIF with the volume of interest (VOI) placed over the heart (method A) compared with AIF. **(B,E)** The IDIF with the VOI set over abdominal vena cava/aorta region with a cuboid (method B) compared with AIF. **(C,F)** the IDIF with the VOI delineated with 1 × 1 × 1 mm voxels on five consecutive slices over the abdominal vena cava/aorta region (method C) compared with AIF. Different dots represent different brain regions (*n* = 9) in all mice (*n* = 5).

As seen in Bland–Altman plots (Figure 4), according to the comparison between AIF and IDIF over heart (method A) (Figures 4A,D), 0% (0/45) and 4.44% (2/45) difference dots were located outside of the 95% limits of agreement by Logan and 2TCM analysis, respectively; in comparison between AIF and method B, 2.22% (1/45) difference dots (Figures 4B,E) were located out of the 95% limits of agreement with both Logan and 2TCM analysis; as for method C, 2.22% (1/45) and 6.67% (3/45) difference dots (Figures 4C,F) were located out of the limits by Logan and 2TCM analysis, respectively. Moreover, based on the comparison between AIF and method A (Figures 4A,D), the ratios of the maximum difference to the mean within the 95% limits of agreement were 25.01% (0.1849/0.7390) and 25.26% (0.1860/0.7363) by Logan and 2TCM analysis, respectively. However, ratios were higher in comparison between AIF and other input functions (methods B and C): 39.33% (0.2856/0.7261), 37.27% (0.2694/0.7227), 38.42% (0.2865/0.7457), and 44.21% (0.3270/0.7396) in Figures 4B,C,E,F, respectively.

Therefore, irrespective of Logan or 2TCM analysis, the best *V*_T_ performance of the IDIFs were found qualitatively with the heart (method A) VOI (Figure 3A).

In detail, the mean *V*_T_ ratios between image-derived and arterial inputs for method A (Table 2) were 1.00 ± 0.17 by Logan and 1.00 ± 0.13 by 2TCM analysis, respectively. Both modeling results were approaching unity. Specific *V*_T_ values of different brain regions obtained with method A by Logan and 2TCM models are given in Supplementary Table 1.

**TABLE 2:** Mean *V*_T_ ratios (IDIF/AIF) obtained from striatum, cortex, hippocampus, thalamus, cerebellum, hypothalamus, amygdala, olfactory bulb, and midbrain for each VOI related image-derived approach in both Logan graphical and 2TCM analysis.

		**Method B**	**Method C**
	**Method A**	**Vena cava/aorta**	**Vena cava/aorta**
	**Heart**	**(cuboid)**	**(1 × 1 × 1 mm)**
	**(mean ± SD)**	**(mean ± SD)**	**(mean ± SD)**
Logan *V*_T_ ratio	1.00 ± 0.17	0.97 ± 0.21	1.02 ± 0.25
2TCM *V*_T_ ratio	1.00 ± 0.13	0.97 ± 0.16	1.02 ± 0.21

**n* = 5. *V*_T_, total distribution volume; VOI, volume of interest; 2TCM, two-tissue compartment model; IDIF, image-derived input function; AIF, arterial input function.*

**FIGURE 5:**
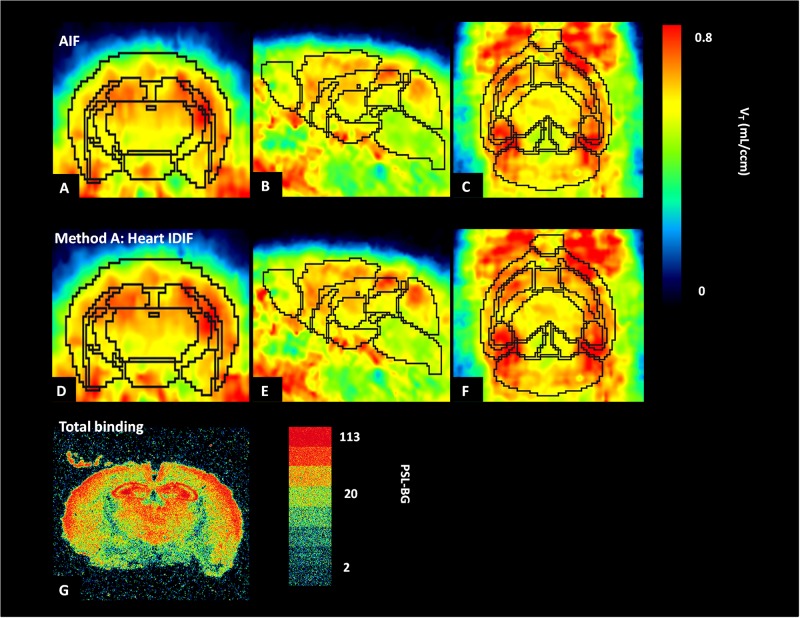
Average parametric images (*n* = 5) of A_1_ adenosine receptors (A_1_ARs) distribution in mice brains calculated by application of Logan plot in PET: coronal plane (left column), sagittal plane (middle column), and horizontal plane (right column) images representing cerebral A_1_AR using the arterial input function (AIF) **(A–C)** and the image-derived input function over the heart (heart IDIF) **(D–F)**. Mouse brain autoradiograph (at 0.99 nM concentration of [^3^H]CPFPX) shows receptor total binding **(G)**.

Figure 5 shows the average parametric images (*n* = 5) of *V*_T_ with Logan plot for both AIF and IDIF over heart (method A). Figures 5A,D present the typical distribution of A_1_AR in mice brains, which is in good accordance to the distribution pattern in autoradiography (Figure 5G).

## Discussion

The main goal of the present study was to establish an optimal [^18^F]CPFPX IDIF which can replace AIF requiring blood sampling in mice. Three non-invasive IDIFs were tested using different VOI-based approaches: (A) VOI placed over the heart (cube, 10 mm); (B) VOI set over abdominal vena cava/aorta region with a cuboid (5 × 5 × 15 mm); and (C) VOI delineated with 1 × 1 × 1 mm voxels on five consecutive slices, also over the abdominal vena cava/aorta region (centered on highest activity spot). We evaluated all the image-derived input results by comparison with standard AIF. We found that method A (VOI placed over the heart) closely follows the AIF. It also showed more accurate, stable and more reliable results than methods B and C in terms of AUC ratios and kinetic modeling of both Logan plot and 2TCM analysis.

As shown in Figures 3, 4, Table 2, and Supplementary Table 1, all results of kinetic modeling analysis with all three image-derived approaches were calculated with both Logan plot and 2TCM. Considering the three different image-derived and the AIFs applied in this study, the Logan-derived *V*_T_ values of a 60-min scan were very similar to those obtained with 2TCM analysis, with a difference of <5% (Supplementary Figure 2). Therefore, in further studies, we propose to directly select Logan analysis for kinetic modeling not only because it is more robust regarding the modeling parameters but also because it is less sensitive than 2TCM modeling analysis toward extraction of the first part of the input curve. Logan analysis based on the AUC of the input function is less sensitive than 2TCM analysis to the initial shape of the input function. The radioactivity concentration of the initial part of whole-blood curve changes rapidly, and consequently, it is difficult to acquire a correct curve from the image data (Zanotti-Fregonara et al., 2011). Under these circumstances, *V*_T_ values are sometimes poorly obtained with 2TCM.

Except from the gold-standard approach of arterial plasma input function and IDIF, the input function for kinetic models can also be acquired by applying a reference region method. When studying brain receptors, cerebellum is used as reference region for some neuroreceptor quantifications. Unfortunately, there often is no reference region for other neuroreceptors, for example, the studies for nicotinic receptors with tracer: (2-[^18^F]fluoro-A-85380) (Zanotti-Fregonara et al., 2012) and mGluR1 with ([^11^C]ITDM) (Bertoglio et al., 2019) in mice.

Different suggestions have been put forward to deal with partial volume as well as spill-out and spill-in effects, respectively. According to Chen et al. (2007) and Kang et al. (2018), the activity *C*_measured_ from VOIs can be considered as a combination of two components: the true radioactivity in the blood vessel and the radioactivity from surrounding regions:

(3) $$C_{\text{measured}}\left(t\right)=\text{PV}\times C_{\text{vessel}}\left(t\right)+\text{SO}\times C_{\text{surrounding}}\left(t\right)$$

where *C*_measured_ (*t*) is the measured radioactivity in the blood obtained from PET, *C*_vessel_ (*t*) is the true radioactivity in the blood vessel, and *C*_surrounding_ (*t*) is radioactivity from surrounding tissues; PV is the determined partial volume correction coefficient, and SO represents the experimental spill-over correction coefficient required to consider the spatial resolution of the small-animal PET scanner and the reconstruction methodology. Lanz et al. (2014) proposed another method for the “spill-out effect” correction of the imaged-derived inputs by a double-exponential function. However, instead of one scaling factor (α), this convolution operation needs four parameter optimizations and a modified Levenberg–Marquardt non-linear regression method. Compared with the above-mentioned studies, we simplified the equations and functions to correct the radioactivity concentration of IDIFs. The main reason for that is that [^18^F]CPFPX rarely showed obvious affinity for myocardium, so that the spill-over effect could be ignored. In our study, because of spill-out and related partial volume effects, the raw image-derived activity of whole blood was always lower than the measured AIF (*p* < 0.05, by *t* test). To correct the above-mentioned effects in IDIFs, an optimal scaling factor (α = 1.77) was calculated (see Eq. 1) and subsequently used for scaling of different IDIFs.

Metabolite correction for IDIF is another important aspect for tracers with high radiometabolite fractions. So far, the majority of studies about the IDIFs prefer to use tracers with negligible metabolism, such as Sari et al. (2017) who discussed image-derived methods for [^18^F]FDG only focusing on partial volume effects and not metabolite correction. For radiotracers with fast metabolism, such as [^18^F]CPFPX, one key part of obtaining IDIFs is the total metabolite correction. In the present study, we not only explored how to solve partial volume effects with a scaling factor (α) but also developed a method of obtaining the total metabolite correction (Eq. 2) of [^18^F]CPFPX for image-derived input approaches. Specifically, after scaling with factor α, the parent curve_(IDIF)_ was obtained by following formulas: parent curve_(IDIF__)_ = whole-blood curve_(IDIF__)_ × (mean parent/whole-blood ratio), mean parent/whole-blood ratio = plasma curve_(IDIF__)_ × total metabolite correction (*t*)/whole-blood curve_(IDIF)_, plasma curve_(IDIF__)_ = 1.67 × whole-blood curve_(IDIF)_.

Although this study required a small amount of blood samples for calculating the scaling factor (α) and the parameters (*a*–*f*) of total metabolite correction equation (Eq. 2), there are still opportunities to achieve completely blood-free IDIFs for acquiring parent curves for future [^18^F]CPFPX or other tracer studies in mice. On the other hand, we can use blood-based methods to optimize the accuracy of the IDIFs. For example, Lanz et al. (2014) tried to decrease the effect of tracer dispersion in blood during the first minute after the [^18^F]FDG bolus injection. They estimated the average scaling parameter using a single blood sample at 1.5 min, which resulted in higher accuracy for the extraction of IDIFs in animals.

There is a potential drawback of the evaluation regarding the AUC ratio. Since the first blood sample was collected 1–2 min after tracer injection, it is possible that the peaks of the arterial blood curves were lower than the peaks of image-derived curves (Figure 2). Taking this into account when using the AUC ratios between image-derived and arterial curves, resulting values would be bigger. This may explain why all AUC ratios in Table 1 were >1. Another limitation of the present study is the relatively low number of mice. However, even though we used only five animals and there were individual differences, we strongly believe in the representativeness of our results. For instance, in Figures 3A–C, although every mouse performed significant correlations (*R*^2^) between arterial and image-derived inputs *V*_T_ values by Logan analysis, there was still no perfect overlay with the line of identity because of the individual slopes and intercepts. However, when we did the average of all five mice, the *V*_T_ values of image-derived inputs got much closer to arterial inputs (Table 2). Notwithstanding these findings, an increased study power will undoubtedly help optimizing the data in further non-invasive input function studies.

In the present study, autoradiography was used for qualitative analysis of A_1_AR with [^3^H]CPFPX. Compared with parametric PET images from [^18^F]CPFPX of arterial and IDIF (Figures 5A,D), the distribution of A_1_AR in autoradiography (Figure 5G) showed the same binding patterns. For example, high concentrations of A_1_AR in cortex, hippocampus, and thalamus were detected by both PET and autoradiography, and the qualitative distribution of A_1_AR labeled with [^3^H]CPFPX (0.99 nM) in coronal sections was similar to previous data of Bailey et al. (2003).

A potential improvement of our image-derived input approach might derive from a closer look at heartbeat affects. Previously, only the left ventricle was used as the region of interest to reduce the motion of heart and improve the precision. Recently, Herraiz et al. (2016) and Verhaeghe et al. (2018) showed that the left ventricle volume can be estimated by means of cardiac gating during PET acquisition. For this purpose, PET raw data were divided into time subsets according to the different phases of the heart activity. Thus, a more accurate measurement and verification of whole-heart image-derived inputs might be obtained in future studies when gated PET imaging will be applied (Locke et al., 2011; Mabrouk et al., 2012).

## Conclusion

The present study provides evidence that a non-invasive IDIF can validly replace the standard AIF in quantification of A_1_AR PET using the fast metabolizing radioligand [^18^F]CPFPX in mice. Furthermore, a VOI over the heart is the best choice to extract the IDIF after comparing different VOI placements regarding image-derived curves, AUC ratios, and kinetic modeling results (*V*_T_).

## Data Availability Statement

The datasets analyzed during the current study are available from the corresponding author on reasonable request.

## Ethics Statement

The animal study was reviewed and approved by the German regional authorities (Landesamt für Natur, Umwelt und Verbraucherschutz) and performed on the basis of the German Animal Welfare Act.

## Author Contributions

DE, AB, and TK designed the study. FW, TK, AO, and DE were responsible for experiments and data collection. XH did analysis and interpretation of data, and drafted and revised the manuscript. DE, TK, SB, and AD made contribution to the data interpretation. JE and BN contributed with tracer synthesis. All authors critically revised the manuscript and approved the final version.

## Conflict of Interest

The authors declare that the research was conducted in the absence of any commercial or financial relationships that could be construed as a potential conflict of interest.
