# Supplementary material for: Image-Derived Input Functions for Quantification of A1 Adenosine Receptors Availability in Mice Brains Using PET and [18F]CPFPX
Source: Front Physiol. 2020 Jan 29;10:1617. doi: 10.3389/fphys.2019.01617 (PMC7000659; doi:10.3389/fphys.2019.01617)
Supplement: Supplementary file 1 [file Data_Sheet_1.pdf]

Supplementary Material

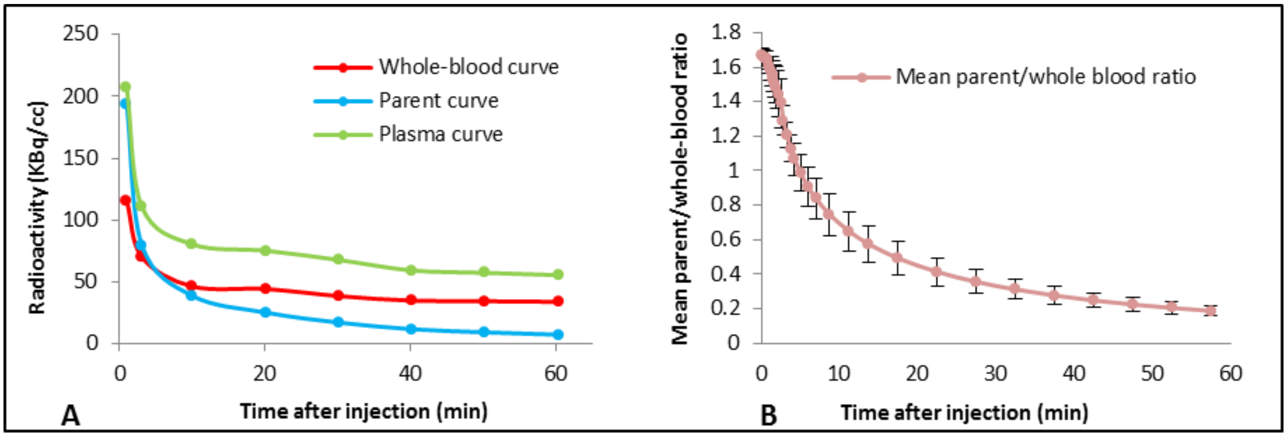

Suppl. Fig. 1 (A) The concentration of radioactivity in whole blood, plasma and parent radioligand in plasma over 60 minutes in arterial blood samples of [^{18}F]CPFPX in a representative mouse. (B) Mean parent / whole-blood ratio = (plasma / whole-blood ratio) × total metabolite correction, n = 5. Parent (of plasma) = plasma × total metabolite correction (the correction should between 1 to around 0.18); plasma = whole-blood × 1.67 (plasma activity showed a stable percentage of whole blood activity), therefore the mean ratio (parent / whole-blood) should between 0.3 to 1.67 after calculation.

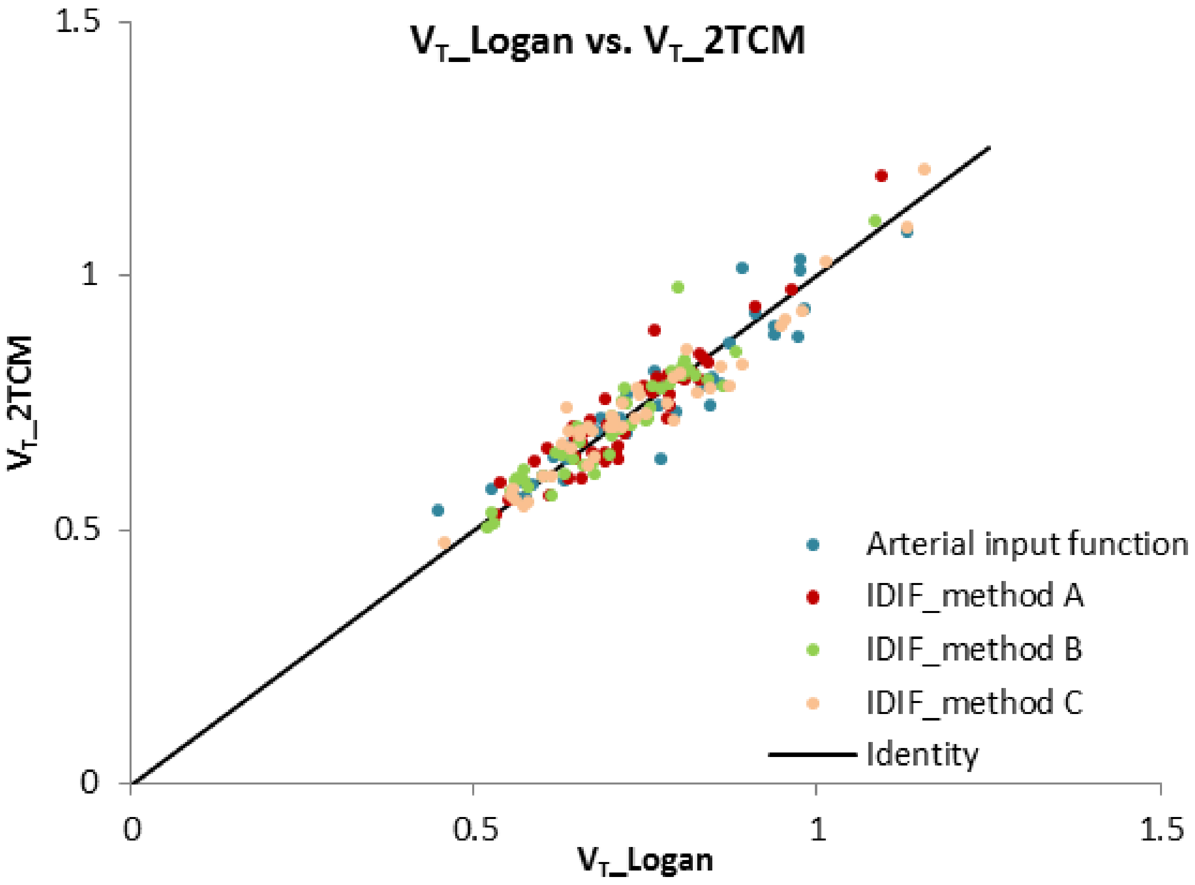

Suppl. Fig. 2 Correlation between distribution volume (V_T) of the 2TCM and Logan analysis by using different image-derived input functions (IDIFs) and arterial input function (AIF) in different brain regions (n = 9) of mice (n = 5).

Suppl. Table 1. The V_T values of the image-derived input with the VOI placed over the heart (method A) and AIF. All V_T values were obtained from different brain regions with the Logan and 2TCM models.

	**Logan V_T values**		**2TCM V_T values**	
	**Method A:** Heart	**AIF**	**Method A:** Heart	**AIF**
striatum	0.71 ± 0.07	0.73 ± 0.14	0.71 ± 0.07	0.70 ± 0.12
cortex	0.75 ± 0.07	0.78 ± 0.12	0.74 ± 0.05	0.78 ± 0.13
hippocampus	0.75 ± 0.04	0.77 ± 0.14	0.75 ± 0.03	0.76 ± 0.12
thalamus	0.65 ± 0.06	0.66 ± 0.13	0.62 ± 0.05	0.66 ± 0.10
cerebellum	0.73 ± 0.06	0.75 ± 0.09	0.73 ± 0.06	0.78 ± 0.12
hypothalamus	0.76 ± 0.07	0.77 ± 0.11	0.78 ± 0.06	0.75 ± 0.07
amygdala	0.77 ± 0.06	0.80 ± 0.14	0.76 ± 0.10	0.78 ± 0.12
olfactory bulb	0.84 ± 0.19	0.85 ± 0.18	0.87 ± 0.22	0.84 ± 0.18
midbrain	0.60 ± 0.05	0.62 ± 0.11	0.60 ± 0.04	0.64 ± 0.10

n = 5

V_T total distribution volume, *VOI* volume of interest, *AIF* arterial input function, *2TCM* two-tissue compartment model
